# The production of ultrahigh molecular weight xanthan gum from a *Sphingomonas* chassis capable of co‐utilising glucose and xylose from corn straw

**DOI:** 10.1111/1751-7915.14394

**Published:** 2024-01-16

**Authors:** Mengmeng Wu, Zhuangzhuang Shi, Yue Ming, Yufei Zhao, Ge Gao, Guoqiang Li, Ting Ma

**Affiliations:** ^1^ Key Laboratory of Molecular Microbiology and Technology, Ministry of Education, College of Life Sciences Nankai University Tianjin China

## Abstract

Corn straw is an abundant and renewable alternative for microbial biopolymer production. In this paper, an engineered *Sphingomonas sanxanigenens* NXG*‐P*_916_ capable of co‐utilising glucose and xylose from corn straw total hydrolysate to produce xanthan gum was constructed. This strain was obtained by introducing the xanthan gum synthetic operon *gum* as a module into the genome of the constructed chassis strain NXdPE that could mass produce activated precursors of polysaccharide, and in which the transcriptional levels of *gum* genes were optimised by screening for a more appropriate promoter, *P*_916_. As a result, strain NXG*‐P*_916_ produced 9.48 ± 0.34 g of xanthan gum per kg of fermentation broth (g/kg) when glucose was used as a carbon source, which was 2.1 times improved over the original engineering strain NXdPE::*gum*. Furthermore, in batch fermentation, 12.72 ± 0.75 g/kg xanthan gum was produced from the corn straw total hydrolysate containing both glucose and xylose, and the producing xanthan gum showed an ultrahigh molecular weight (UHMW) of 6.04 × 10^7^ Da, which was increased by 15.8 times. Therefore, the great potential of producing UHMW xanthan gum by *Sphingomonas sanxanigenens* was proved, and the chassis NXdPE has the prospect of becoming an attractive platform organism producing polysaccharides derived from biomass hydrolysates.

## Graphical abstract

Corn straw is an abundant and renewable alternative for microbial biopolymer production. To facilitate the bioconversion into polysaccharide, a chassis Sphingomonas sanxanigenens NXdPE capable of co‐utilizing glucose and xylose from corn straw total hydrolysate and mass‐producing activated precursors of polysaccharide was constructed. Then a xanthan‐producing engineered strain NXG‐P916 was obtained by introducing the synthetic module of xanthan into the genome and optimizing the transcriptional level of operon gum. What’s more, an ultrahigh molecular weight (UHMW) xanthan of 6.04 × 107 Da was first produced, which was 15.8 times as much as the wild‐type xanthan. Therefore, the great potential of producing UHMW xanthan from lignocellulosic biomass was proved in this work, and the chassis NXdPE has a prospect of becoming an attractive platform organism producing polysaccharides derived from biomass hydrolysates.

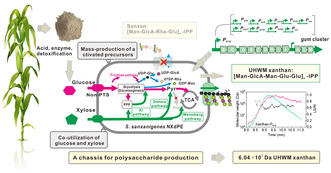

## INTRODUCTION

Lignocellulosic biomass is the most abundant and renewable organic material in the biosphere; for example, corn straw generates over 200 million tonnes each year (Cho et al., 2020; Rastogi & Shrivastava, 2017). This biomass waste can cause serious environmental problems if it is not disposed of properly. Therefore, developing an eco‐friendly and effective strategy for using and managing lignocellulosic waste is critical. Lignocellulose is composed of cellulose, hemicellulose and lignin, and it can be converted into glucose and xylose through alkaline, acid and enzymatic hydrolysis (Sindhu et al., 2016). The ability of microorganisms to ferment both glucose and xylose into high‐value products is of importance for an economically feasible process (Zhao et al., 2008). One problem with the use of mixed sugars is carbon catabolite repression (CCR) (Fujiwara et al., 2020; Kremling et al., 2015). Many engineered strains have been constructed to relieve CCR and enhance xylose metabolism (Fu et al., 2017; Lee et al., 2015), such as the knockdown of the phosphotransferase system (PTS) (Gonzalez & Antoniewicz, 2017). However, this modification of metabolism may cause decreases in productivity and yield (Wang et al., 2018). Hence, for an efficient and complete conversion of glucose and xylose, the microorganism that natively co‐utilises mixed sugar may be an effective bioproduct‐producing chassis.

As a kind of natural polymer microbial polysaccharides have been used as thickeners, adhesives, probiotics, gelling agents and stabilisers in the food, medical, cosmetic and petroleum industries and as emulsifiers, biosorbents and bioflocculants in the environmental sector (Freitas et al., 2017). However, when compared with synthetic polymers, natural polysaccharides still represent only a small fraction of the current polymer market, mostly due to their costly production processes, and fermentation medium represents almost 30% of the cost for microbial fermentation (Öner, 2013). The utilisation of lignocellulose as a carbon source will effectively maximise the cost‐effectiveness of polysaccharide production. However, glucose‐rich enzymatic hydrolysate and xylose‐rich acid/alkali hydrolysate were always used separately to produce polysaccharides in most reports (Abdeshahian et al., 2020; Jazini et al., 2017; Jesus et al., 2014; Wang et al., 2014). *Sphingomonas sanxanigenens* NX02 could naturally co‐utilise glucose and xylose from corn straw total hydrolysate to produce the polysaccharide Sanxan (Wu et al., 2021), which was a promising alternative in the food, cosmetic and petroleum industries because of its excellent gel‐forming, emulsifying and thickening properties (Wu et al., 2017). The tetrasaccharide repeat unit structure of Sanxan was (1→4)‐D‐Mannose (Man) (1 → 4)‐D‐Glucuronic acid (GlcA) (1 → 3)‐L‐Rhamnose (Rha) (1 → 3)‐D‐Glucose (Glu) (1 → Isopentenyl pyrophosphate (IPP)), which was a sphingan with a distinctly different primary structure from gellan, welan and diutan (Huang et al., 2016). Three coexisting xylose metabolic pathways: XI (xylose isomerase), Weimberg and Dahms were found in the genome of strain NX02, which promoted the effective utilisation of xylose (Wu et al., 2021). Moreover, the biosynthetic flux of active sugar precursors was large since strain NX02 was a polysaccharide‐producing strain. Therefore, strain NX02 could be an attractive chassis for polysaccharide synthesis from lignocellulose wastes.

Xanthan gum, as a typical extracellular polysaccharide produced by *Xanthomonas campestris*, has been widely used in the food, cosmetic, agriculture and petroleum industries (Berninger et al., 2021; Martins et al., 2018). Its primary structure is a cellulose‐like backbone with a trisaccharide side chain of two mannoses and one glucuronate residue (Jansson et al., 1975; Melton et al., 1976; Smith et al., 1981). The molecular weight ranges from 2.0 × 10^6^ to 2.0 × 10^7^ Da, which could be influenced by the strain and variations of the fermentation conditions (García‐Ochoa et al., 2000). The higher the molecular weight, the higher the viscosity (Galván et al., 2013; Milas et al., 1985), which will result in superior rheological properties. Compared with low molecular weight xanthan gum that could be obtained through enzymatic and chemical methods (Hu et al., 2019; Xiong et al., 2013), ultrahigh molecular weight xanthan gum (greater than 2.0 × 10^7^ Da, UHMW xanthan gum) may only be obtained by the fermentation of engineered strains, but there have been no related reports about this product or strain till now.

The biosynthetic pathway of xanthan gum follows the Wzx/Wzy‐dependent pathway, and the genes required for the synthesis of sugar precursors, polymerisation and exportation are clustered in a 16 ‐kb operon *gum* containing 12 open reading frames (*gumB* to *gumM*) (Becker et al., 1998). Additionally, there are two promoters in operon *gum*: A promoter, that is, *P*_*gum*_, located upstream of the first gene *gumB*, and a second promoter was identified upstream of *gumK* (Becker et al., 1998; Katzen et al., 1996). For xanthan gum fermentation, the major carbon sources in batch culture are glucose, sucrose and starch (Becker et al., 1998; García‐Ochoa et al., 2000). When cultured in a medium containing glucose and xylose, the strain always utilised glucose as a preferred carbon source (Zhang & Chen, 2010). Recently, xylose‐rich acid hydrolysate of broomcorn stem (Zahra et al., 2018), alkaline hydrolysate of corn cob liquor (Jesus et al., 2014) and glucose‐rich enzymatic hydrolysate of rice straw (Jazini et al., 2017) have been utilised as carbon sources to produce xanthan gum. Additionally, other different lignocellulose partial hydrolysates have also been used as carbon sources to produce xanthan gum, such as alkaline hydrolysed corncob (Jesus et al., 2023), acid hydrolysed *Melaleuca alternifolia* residue (Li et al., 2022), self‐hydrolysed coconut shells and cocoa husks under sterilisation conditions (da Silva et al., 2018), acid hydrolysed orange peels (Mohsin et al., 2018), and enzymatically hydrolysed potato crop residues (Soltaninejad et al., 2022), but the full utilisation of total hydrolysate rich in both glucose and xylose to produce xanthan gum has not been reported.

There are two ways to efficiently convert lignocellulose, such as corn straw, into xanthan: modification of the original strain to enhance xylose metabolism and introducing the xanthan gum synthetic pathway as a module into a chassis strain that could naturally utilise mixed sugars. Herein, xanthan was successfully produced in a chassis based on strain NX02, and its yield was significantly improved by optimisation of the transcriptional level of operon *gum*. What is more, the product was an ultrahigh molecular weight xanthan gum. This work demonstrates the effectiveness of the chassis strain for polysaccharide production and promotes the conversion of corn straw total hydrolysate to UHMW xanthan gum.

## EXPERIMENTAL PROCEDURE

### Preparation of corn straw total hydrolysate

Corn straw, as a recalcitrant plant biomass, is composed of cellulose, hemicellulose and lignin, which are all difficult to directly utilise by microorganisms. Herein, corn straw was collected, dried at room temperature and milled to a particle size no >0.425 mm (40 mesh), followed by acid and enzymatic hydrolysis (Jazini et al., 2017; Xu et al., 2010). Activated charcoal (100 mesh, Aladdin Reagent Co., Ltd, Shanghai, China) was added to discolour and remove the impurities like phenolics and proteins in the total hydrolysate and removed by centrifugation or filtration system (Kumar et al., 2019). The detailed methods were presented in our previous report (Wu et al., 2021). Finally, the total hydrolysate containing 52.6 g/L glucose, 15.1 g/L xylose and 1.8 g/L arabinose was obtained and then stored at 4°C.

### Strains, plasmids and media

The bacterial strains and plasmids used in this work are listed in Table 1. *Sphingomonas sanxanigenens* NX02 (CGMCC 10150) and its derivative strains were cultured at 30°C for 24 h on NKG (NK medium with 15 g/L glucose; NK medium (per litre): glucose 15.0 g, peptone 5.0 g, beef powder 3.0 g, yeast extract 1.0 g and agar 15.0 g, pH 7.0) and NKS medium (NK medium with 8% sucrose). Ten percent (v/v) NKG culture was inoculated into the fermentation medium consisting of (per litre): carbon sources 40.0 g, yeast extract 0.2 g, K_2_HPO_4_ 1.2 g, NaNO_3_ 2.0 g, CaCO_3_ 1.0 g, FeSO_4_ 0.005 g, NaCl 0.4 g, and MgSO_4_ 0.5 g (pH 7.5) (Wu et al., 2016). *Escherichia coli* S17, as the donor in the conjugal transfer process, carrying different plasmids, was grown in LB medium at 37°C. *Xanthomonas campestris* CGMCC 15155 was cultured at 30°C for 24 h on NKG medium. The carbon sources are glucose, xylose, mixed sugars or corn straw total hydrolysate.

**TABLE 1:** Bacterial strains and plasmids used in this work

Strain or plasmid	Genotype or phenotype	Source or reference
Strains		
*Sphingomonas sanxanigenens* NX02	Wild‐type strain, EPS^+^, PHB^+^	This work
*Sphingomonas sanxanigenens* NXdP	PHB‐defective strain from the wild‐type strain NX02, PHB^−^	Wu et al. (2018)
*Sphingomonas sanxanigenens* NXdPE	Engineered strain, EPS^−^, PHB^−^	This work
NXdPE::*gum*	Gene cluster *gum* was inserted into the genome of strain NXdPE, xanthan^+^, Sanxan^−^, PHB^−^	This work
NXdPE (pBBR*gum*)	Strain NXdPE harbouring plasmid pBBR*gum*, xanthan^+^, Sanxan^−^, PHB^−^	This work
NXdPE (pBC*P*_*n*_ *rfp*)	Strain NXdPE harbouring plasmid pBC*P*_*n*_ *rfp*, ‘*P*_*n*_’ refers to *P*_828_, *P*_916_, *P*_1218_, *P*_4936_, *P*_5194_, *P*_5217_, *P*_5218_, *P*_5286,_ *P*_*tac*_, *P*_*lac*_	This work
NXdPE::*P*_*n*_ *gum*	The promoter of *gum* in strain NXdPE::*gum* was replaced by *P*_*n*_	This work
NXdPE::*P*_916_ *gum* (pBT*gumBCDE*)	Strain NXdPE::*P*_916_ *gum* harbouring plasmid pBT*gumBCDE*	This work
*Xanthomonas campestris* CGMCC 15155	Wild‐type strain, xanthan^+^	This work
*Escherichia coli* S17	RecA thi pro hsdR*−* M+ RP4, Sm^R^ Amp^R^ Kan^R^	This work
Plasmids		
pBBR1mcs‐2	Broad host range vector, kan^R^	Kovach et al. (1995)
pLO3	Suicide vector, tet^R^	Lenz & Friedrich (1998)
pBC	Broad host range vector without promoter, kan^R^	This work
pBT	Broad host range vector with promoter *P*_*tac*_, kan^R^	Wu et al. (2019)
pLO3gum	Suicide vector harbouring upstream and downstream homologous arms and replacement segments	This work
pBBR*gum*	pBBR1msc‐2 derivative expressing gene cluster *gum*	This work
pBT*gumBCED*	pBBR1msc‐2 derivative expressing *gumBCDE*	This work
pBC*P*_*n*_ *rfp*	pBC derivative expressing *P*_***n***_ *rfp*	This work

The broad host range constitutive expression pBBR1mcs‐2 and its derived plasmids pBC (deleting the promoter *P*_*lac*_ sequence) and pBT (replacing *P*_*lac*_ promoter with the strong *P*_*tac*_ promoter) were used for gene overexpression (Wu et al., 2019). The suicide vector pLO3 was used for gene knock‐out, knock‐in, and replacement (Lenz & Friedrich, 1998). The plasmids pLO3*gum* and pBBR*gum* were constructed using the Gibson Assembly method, and other plasmids were constructed using restriction enzymes and ligases. Primers for construction of all plasmids are shown in Table S1. Antibiotics were used at the following concentrations (μg/mL): tetracycline (Tc; 10), kanamycin (Km; 25) and Chloramphenicol (Cm; 25).

### Genetic manipulation for the construction of engineered strains

Genes were knocked out, knocked in and replaced by double‐crossover homologous recombination. The upstream and downstream flanking sequences of the target gene, together with gene fragments that need to be knocked in or replaced, were ligated into vector pLO3. The recombinant suicide plasmids were transformed into *E. coli* S17 and transferred to the *S. sanxanigenens* strain using biparental conjugational transfer at 30°C for 12 h on NKG medium. The single crossover mutants were selected on NKG medium containing 10 μg/mL Tc and 25 μg/mL Cm, while double crossover stains were isolated on NKS medium with 25 μg/mL Cm, followed by PCR screening using the verification primers (Wu, Huang, et al., 2017). Similarly, the expression plasmids were transformed into *S. sanxanigenens* strains by biparental conjugational transfer, then verified by PCR. The primers for genes *ssH*, *ssQ* and the operon *ssGCD* knockout, identification and gene overexpression are shown in Table S1.

### Chain‐specific transcriptome sequence and reverse transcription quantitative PCR (RT‐qPCR)

Chain‐specific transcriptomes of strain NX02 cultured in the carbon sources of glucose and xylose were investigated to screen out internal promoters with different strengths. The strain was cultivated for 24 h on NK medium with glucose and xylose, respectively. The crude total DNA‐free RNA was extracted and reverse‐transcribed using the RNAprep Pure Cell/Bacteria Kit (Tiangen, China) and RNAiso Plus (Takara, Dalian, China). Sequencing was performed at Novogene Bioinformatics Technology Co. Ltd. (Beijing, China). Seven constitutive‐expression genes with separate transcripts in glucose and xylose were chosen according to the different FPKM (fragments per kilobase of exon per million mapped reads) values and gene structure analysis. The promoter sequences of these genes were predicted using BDGP Neural Network Promoter Prediction V2.2 (Reese, 2001), and the detailed sequences of these promoters and annotations of related genes are shown in Table S2. All the selected promoters were ligated with the red fluorescent protein marker gene *rfp* into plasmid pBC to obtain plasmid pBC*P*_*n*_ *rfp* (*P*_*n*_ refers to the selected promoters) and then transformed into the chassis through biparental conjugational transfer. The chassis (pBC*P*_*n*_ *rfp*) was cultivated on NKG medium. When growing to 24 h, cells were collected for the analysis of promoter strength by the fluorescence intensity of protein RFP and the transcriptional level of gene *rfp*. The plasmid pBC was a blank control. Additionally, the relative transcriptional levels of *gum* genes in different engineered strains were also measured by RT‐qPCR. Engineered strains with promoter‐*gum* inserted into their genomes or harbouring plasmid pBT*gumBCDE* were cultivated on the fermentation medium with glucose. When growing to 60 h, xanthan gum accumulates rapidly at this time, cells were collected, and the crude total DNA‐free RNA was extracted and reverse‐transcribed for quantitative analysis. The relative expression analysis of *gum* genes was performed by RT‐qPCR with a MyiQ™ two‐colour real‐time PCR detection system (BIO‐RAD laboratories) with the Bestar® SybrGreen qPCR mastermix (DBI, Bioscience Inc., Germany). The endogenous reference gene was 16S rRNA. The primers used for RT‐qPCR are listed in Table S1. Standard deviations were calculated from three PCR replicates, and the relative abundance of the genes was determined using the comparative Ct method.

### Fermentation of engineered strains to produce xanthan gum

Cultivation of engineered strains was performed using shake flasks (30°C, 200 rpm, 96 h) and a 5 L quadruple autoclavable fermenter (Shanghai Bailun Biotechnology Co., Ltd., Shanghai, China). The temperature and pH were maintained at 30°C and 7.0, respectively, and the detailed batch fermentation method was shown in our previous work (Wu et al., 2021).

### Analytical methods

#### Determination of dry cell weight (DCW) and xanthan gum yield

Xanthan gum was secreted outside the cell, and it can be separated from the cell using dilution, centrifugation and precipitation procedures. Ten grams of fermentation broth from different strains were collected, diluted ten‐fold with deionised water and then centrifuged at 12,000 *g* for 20 min to separate the supernatant xanthan solution and cell pellet. The cell pellet was dried to a constant weight to analyse its DCW. And xanthan was precipitated by a three‐fold volume of non‐aqueous industrial alcohol, then weighed to calculate its yield after drying at 90°C for 3 h. The unit g/kg represented the yield of DCW and xanthan gum per kilogram of fermentation broth. Herein, all measurements were repeated at least three times.

#### Contents determination of glucose and xylose

The contents of glucose, xylose and arabinose in corn straw total hydrolysate and fermentation broth were determined using high pressure liquid chromatography (HPLC, Agilent 1100, USA) equipped with a refractive index detector and a 6.5 × 300 mm Waters Sugar‐Pak I column (Waters, Milford, MA, USA). The eluent was 50 mg/L calcium disodium EDTA at a flow rate of 0.5 mL/min (Wu et al., 2021). The standard curves were measured using different concentrations of glucose, xylose and arabinose (15, 30, 45, 75, and 100 mg/L).

#### The primary structure analysis of xanthan gum produced by an engineered strain

Infrared spectra of the polysaccharide produced by the engineered strain were recorded with a 560E.S.P (Nicolet, USA) FT‐IR spectrometer in a KBr pellet. The weight average molar mass (MW) of the polysaccharides was determined using size‐exclusion chromatography combined with multi‐angle laser light scattering (SEC‐MALS) (Huang et al., 2016). The monosaccharide composition of polysaccharide was determined using HPLC equipped with a refractive index detector and a 6.5 × 300 mm Waters Sugar‐Pak I column (Waters, Milford, MA, USA). Samples (5 mg) were hydrolysed with 2.0 mL of 2.0 M trifluoroacetic acid (TFA) at 110°C for 12 h and the precipitate after volatilisation was remelted into the water to filter and inject for HPLC analysis. The eluent was 50 mg/L EDTA calcium sodium at a flow rate of 0.5 mL/min. The column was maintained at 85°C.

#### Fluorescence intensity measurement of protein RFP

The fluorescence intensity of protein RFP was used to characterise the promoter strength of internal promoters, and it was measured by an automatic microplate reader (Enspire, PerkinElmer LLC). Strains were centrifuged at 2000 *g* for 2 min and resuspended in 200 μL of phosphate‐buffered saline (PBS). The relative fluorescence intensity was calculated as the ratio of the RFP fluorescence (excitation and emission wavelengths are 584 and 607 nm, respectively) to the absorbance of the cell suspension at a wavelength of 600 nm, and the mean and SD from three biological experiments were calculated.

## RESULTS AND DISCUSSION

### Construction of a chassis strain NXdPE that co‐utilised glucose and xylose

**FIGURE 1:**
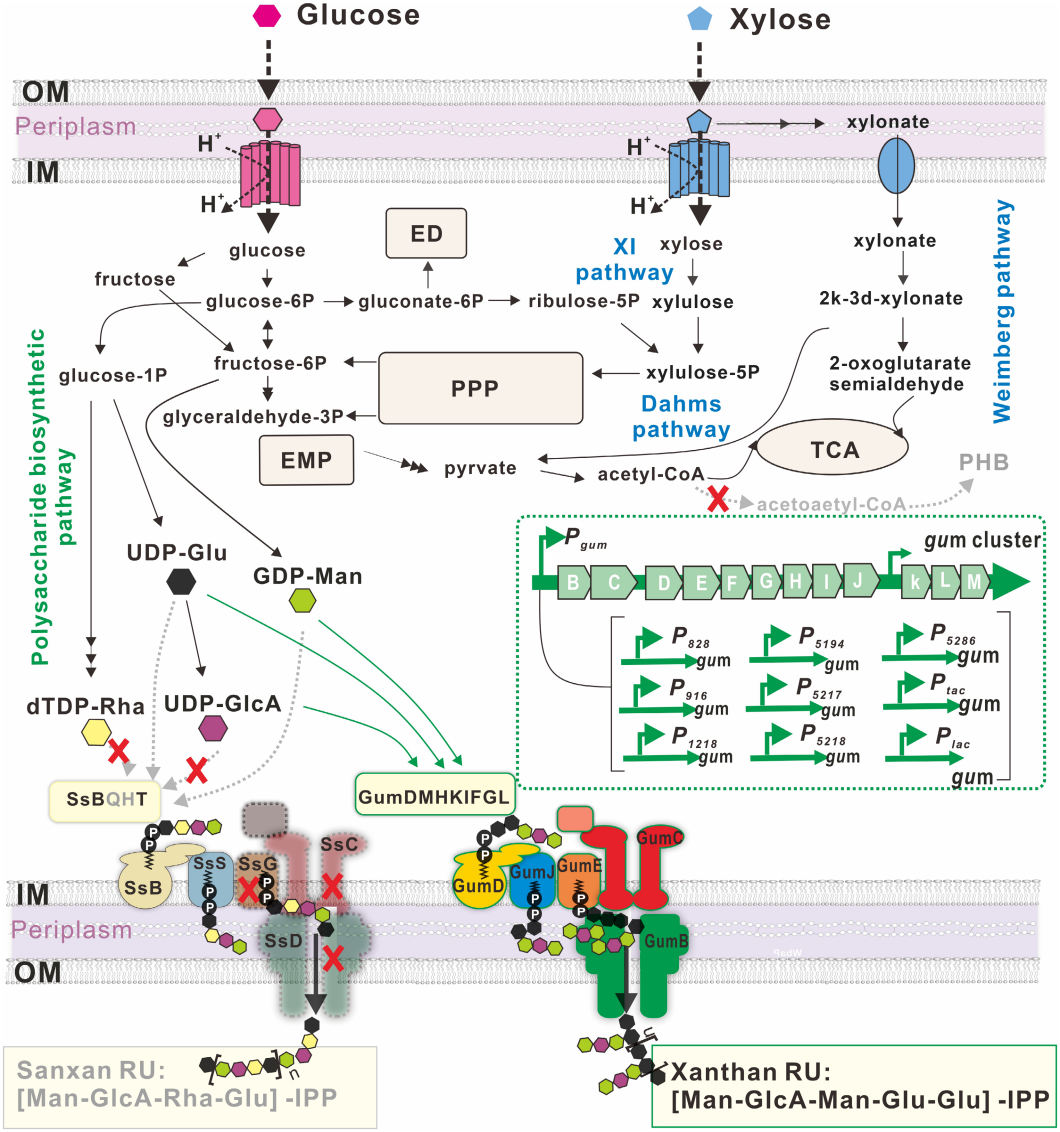
Overview of the engineered pathway for the synthesis of xanthan gum from glucose and xylose in *Sphingomonas sanxanigenens*. Black‐coloured arrows indicate native metabolic pathways; grey metabolites and grey‐coloured arrows with dash lines indicate the critical blocked steps for PHB and polysaccharide biosynthesis; green‐coloured thin arrows indicate the introduced pathways for xanthan gum synthesis; green‐coloured thick arrows indicate the different promoters; red crosses indicate the knock‐out step. dTDP‐Rha, dTDP‐rhamnose; ED, Entner‐Doudoroff pathway; EMP, glycolytic pathway; GDP‐Man, GDP‐mannose; IM, inner membrane; OM, outer membrane; PPP, pentose phosphate pathway; RU, repeating unit; TCA, tricarboxylic acid cycle; UDP‐GlcA, UDP‐glucuronic acid; UDP‐Glu, UDP‐glucose.

**FIGURE 2:**
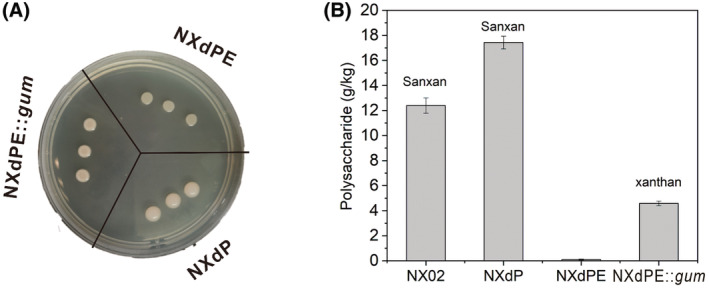
Cultivation of strain NXdP, NXdPE and NXdPE::*gum* (A) and production of Sanxan and xanthan gum in different strains (B).

*Sphingomonas sanxanigenens* NX02 was a wild‐type strain that could coproduce the polysaccharide Sanxan and poly(R)‐3‐hydroxybutyrate (PHB) through the co‐utilisation of glucose and xylose (Figure 1) (Wu et al., 2016, 2021). After the markerless knockout of six crucial PHB biosynthetic gene *phbB* copies and non‐rational plasma mutagenesis, a PHB‐defective mutant NXdP was obtained (Wu et al., 2018). Based on strain NXdP, five genes for Sanxan synthesis were deleted to construct a chassis strain that could not produce the exopolysaccharide Sanxan, named NXdPE (Figure 1). Glycosyltransferases SsH and SsT, Flipase SsS and polymerisation and export protein SsG, SsC and SsD were all specific for the unique tetrasaccharide repeating unit structure of Sanxan (Wu, Huang, et al., 2017). Herein, gene *ssH*, *ssQ* and the operon *ssGCD* were markerless knockouts in turn. SsH, which catalyses the connection of β‐D‐glucuronic acid to α‐L‐Rhamnose (Wu, Huang, et al., 2017), was first deleted, and the resulting mutant strain could not produce Sanxan. SsQ, as the second deleted glycosyltransferase, transfers rhamnose from dTDP‐L‐Rhamnose to IPP‐glucose, and its inactivation could prevent the formation of the disaccharide intermediate L‐Rha (1 → 3)‐D‐Glu (1 → IPP). Finally, the operon *ssGCD*‐encoding membrane protein complex was deleted completely to release the membrane space. As a result, the phenotypes of strains NXdP and NXdPE were different. The single colony of strain NXdPE was small and flat (Figure 2A). Fermentation experiments also showed that the yield of Sanxan from the engineered strain NXdP was much higher than the wild‐type strain NX02, while NXdPE could not produce polysaccharide (Figure 2B).

The priming glycosyltransferase SsB, annotated as glucose‐1‐isoprenylphosphate transferase that could transfer glucose‐1‐phosphate from UDP‐glucose to a lipid carrier comprising the C55‐isoprenylphosphate carrier (IP), was not deleted because it existed in the synthesis of most glucose‐initiated polysaccharides. Genes responsible for precursor synthesis were also not modified in strain NXdPE. Therefore, chassis strain NXdPE still has the potential to mass‐produce activated precursors, then assemble, polymerise and export other polysaccharides, such as xanthan gum, gellan, hyaluronic acid and other glycosaminoglycans.

Additionally, the chassis NXdPE have three characteristics that are conducive to co‐utilise glucose and xylose: Coexistence of three xylose metabolic pathways (XI, Weimberg, and Dahms pathway), incomplete phosphoenolpyruvate‐dependent phosphotransferase system and reinforced fructose metabolism (A two‐step reaction from glucose to fructose to fructose‐6‐phosphate catalysed by glucose isomerase and fructokinase) (Wu et al., 2021). Therefore, the chassis NXdPE will be an attractive platform organism to produce polysaccharides and other bio‐based products derived from agricultural waste hydrolysate rich in both glucose and xylose.

### Construction of xanthan‐producing strains

To stably produce xanthan, the operon *gum* was knocked into the genome of the chassis. The insertion locus was gene *orf5016*, which was annotated as a glycosyltransferase, and its deletion did not affect strain growth and polysaccharide synthesis, according to our previous work (Wu, Huang, et al., 2017). Operon *gum* containing two promoters upstream of *gumB* and *gumK* as an expression module was divided into three segments of similar size: *gumBCDE* (5.3 kb), *gumEFGHI* (4.6 kb) and *gumJKLM* (5.2 kb), together with the upstream and downstream fragments of insertion locus of gene *orf5016*, ligated into vector pLO3, respectively. These three segments were knocked in the genome in turn through homologous recombination, and the engineered strain NXdPE::*gum* was successfully constructed. In the operon *gum*, genes *gumDMHKIFGL* are annotated as glycosyltransferases and acetyltransferases, among them, GumD as the priming glycosyltransferase is the isoenzyme of SsB; genes *gumFGL* encode acetyltransferase, and genes *gumJ*, *gumE*, *gumC* and *gumB* encode flipase, polymerase, polysaccharide co‐polymerase and export lipoprotein, respectively, which are all membrane proteins and responsible for the polymerisation and export of xanthan gum (Becker et al., 1998; Figure 1). The phenotype of the single colony was much stickier and larger than that of the chassis NXdPE (Figure 2A), and shaking flask fermentation demonstrated that strain NXdPE::*gum* could produce xanthan gum, and the yield was 4.58 ± 0.18 g/kg (Figure 2B). The low yield of xanthan gum might be caused by the low compatibility between the xanthan expression module and the chassis NXdPE, especially the membrane proteins GumBCDEJ.

### Optimisation of transcriptional level to improve xanthan gum yield

**FIGURE 3:**
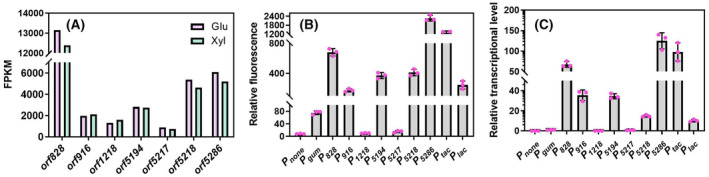
Characterisation of endogenous promoters of strain NX02. (A) The FPKM values of seven selected genes of strain NX02 cultured in glucose and xylose, respectively; (B) the relative fluorescence intensity of protein RFP in strain NXdPE (pBC*P*_*n*_ *rfp*) cultured in glucose medium; (C) the transcriptional level of gene *rfp* in strain NXdPE (pBC*P*_*n*_ *rfp*) cultured in glucose medium.

To increase the yield of xanthan gum, the transcriptional level of the synthetic module from strain NXdPE::*gum* was optimised by replacing promoters with different intensities. The FPKM values of seven selected promoters were gradually increased (Figure 3A). Among them, the FPKM value of gene *orf828* was the highest, while those of gene *orf1218* and *orf5217* were low. These promoters together with promoter *P*_*gum*_, *P*_*tac*_ and *P*_*lac*_ were ligated with gene *rfp* into plasmid pBC, then transformed into strain NXdPE to obtain strain NXdPE (pBC*P*_*n*_ *rfp*). Next, the intensity of these promoters and the blank control *P*_*none*_ of pBC was further determined through the expression of red fluorescent protein RFP and the transcriptional level of gene *rfp* (Figure 3B,C). The differences in the relative fluorescence of RFP and the relative expression level of gene *rfp* were similar in different strains. Furthermore, these promoters were classified into three types: three strong promoters (*P*_828_, *P*_5286_ and *P*_*tac*_; fluorescence/OD_600_ > 600, relative transcription level > 50), four moderate promoters (*P*_916_, *P*_5194_, *P*_5218_ and *P*_*lac*_; 100 < fluorescence/OD_600_ < 600, 10 < relative transcription level < 50), and three weak promoters (*P*_1218_, *P*_5217_, and *P*_*gum*_; fluorescence/OD_600_ < 100, relative transcription level < 10). The intensity of *P*_5286_ was strongest, followed by *P*_*tac*_ and *P*_828_, and the intensity of *P*_1218_ and *P*_5217_ were lower than the control *P*_*gum*_. This trend was slightly different from the FPKM values of the original genes, especially the promoter before gene *orf828*, which might be due to the regulating effect caused by different locations and copies of the promoter. In brief, the intensity of these selected promoters was expressed from high to low (Figures 1 and 3).

**FIGURE 4:**
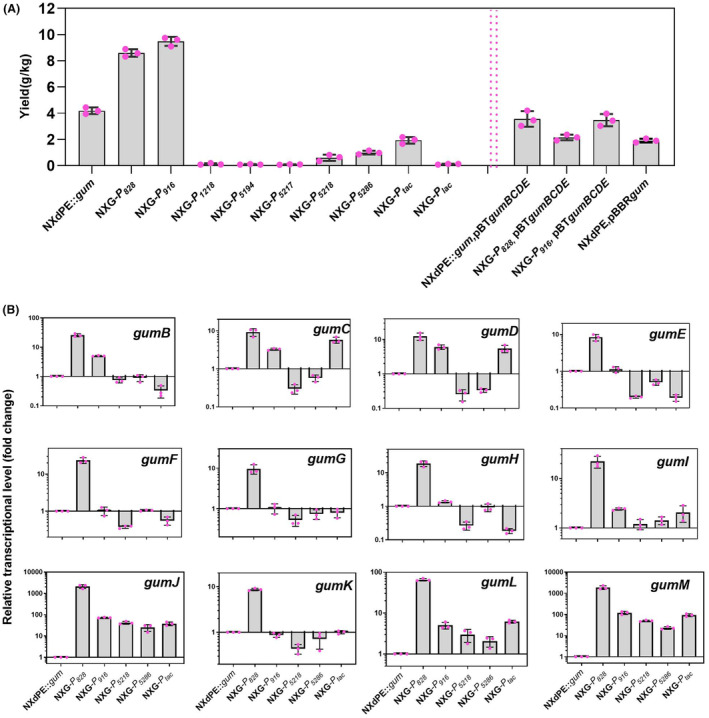
The xanthan gum yields from different engineered strains through promoter optimisation and gene overexpression (A) and the relative transcriptional levels of 12 *gum* genes in six engineered strains (B).

The original promoter *P*_*gum*_ of operon *gum* was replaced respectively by seven native and two commercial promoters to construct nine engineered strains, named NXdPE::*P*_*n*_ *gum* (NXG*‐P*_*n*_ for short, ‘*P*_n_’ indicates promoter). Their xanthan gum yields from shake‐flask fermentation are shown in Figure 4A. NXG*‐P*_916_ produced xanthan gum with the highest yield of 9.48 ± 0.34 g/kg, which was 2.1 times the yield of strain NXdPE::*gum*, followed by the strain NXG*‐P*_828_ with a yield of 8.60 ± 0.29 g/kg. However, the relative fluorescence and transcriptional levels of promoter *P*_828_ were both higher than those of promoter *P*_916_ (Figure 3B,C). Strain NXG*‐P*_5286_ with the strongest promoter only produced 1.00 ± 0.14 g/kg xanthan gum, while strain NXG*‐P*_*tac*_ with a relatively strong promoter *P*_*tac*_ could produce 1.94 ± 0.25 g/kg xanthan gum. Strains NXG*‐P*_5194_, NXG*‐P*_5218_ and NXG*‐P*_*lac*_ with moderate promoters and strains NXG*‐P*_5217_ and NXG*‐P*_1218_ with weak promoters all could hardly produce xanthan gum. This result suggested that the weak promoter was not beneficial to xanthan gum synthesis, and it was not that the stronger the promoter, the more conducive to xanthan gum production. This phenomenon was also found in the biosynthesis of hyaluronic acid (HA) in engineered *Corynebacterium glutamicum*, a moderate strengthening of *hasB* (encoding UDP‐glucose 6‐dehydrogenase oxidises UDP‐glucose to UDP‐glucuronic acid, which is one of the precursors of HA) expression was beneficial to HA production (Cheng et al., 2019). The appropriate promoter will facilitate the synthesis of xanthan gum. Herein, promoter *P*_916_, as a moderate promoter, was the optimal one for xanthan gum production. The significant difference among promoters *P*_828_, *P*_916_ and the other three moderate promoters might be related to their different abilities in driving *gum* transcription. To explain this phenomenon, six xanthan‐producing strains with different promoters (the original promoter *P*_*gum*_, strong promoters *P*_828_, *P*_5286_ and *P*_*tac*_, and moderate promoters *P*_916_ and *P*_5218_) were selected to further analyse the relative transcriptional levels of all 12 *gum* genes; strain NXdPE::*gum* with the original promoter *P*_*gum*_ was a control (Figure 4B).

Compared with strain NXdPE::*gum*, the transcription levels of *gum* genes from strain NXG*‐P*_828_ were all upregulated by 8.4 to 2124.2 times, but in the other strong promoters harbouring strains NXG*‐P*_5286_ and NXG*‐P*_*tac*_, transcriptional levels of only some genes were upregulated. In strain NXG*‐P*_5286_, except for genes *gumJ* and *gumM*, transcription levels of the other 10 genes were downregulated or did not change significantly, while in strain NXG*‐P*_*tac*_, those of genes *gumB*, *gumE* and *gumH* were all dramatically downregulated. In strains harbouring moderate promoters, the transcription levels of genes *gumB*, *gumC*, *gumD*, *gumJ*, *gumL* and *gumM* of strain NXG*‐P*_916_ with the highest xanthan gum yield were all upregulated by 3.3 to 118.5 times, while those of *gum* genes in strain NXG*‐P*_5218_, like strain NXG*‐P*_5286_, were mostly downregulated or did not change significantly. This result was inconsistent with the characterisation of promoter activity, which might be caused by the unreasonable location of the promoter or the low adaptability between promoter and operon. But for promoter *P*_828_, the transcriptional level of the *gum* operon was consistent with the FPKM value of gene *orf828* (Figure 3A), which suggested that promoter *P*_828_ showed more activity on the genome than that of the plasmid. The low transcription levels of most *gum* genes (especially *gumB* and *gumE*) in strains NXG*‐P*_5218_, NXG*‐P*_5286_ and NXG*‐P*_*tac*_ were the key reason for the low xanthan gum yield. The reason why strain NXG*‐P*_828_ with the highest *gum* transcriptional levels showed lower xanthan gum yield than strain NXG*‐P*_916_ might be because the translation levels of 12 genes and the effectiveness of membrane protein machine GumBCDEJ assembly and exact‐running from strain NXG*‐P*_916_ were more conducive to xanthan gum synthesis. Five proteins, GumBCDEJ, could form a precise and critical membrane protein machine for the assembly, polymerisation and export of xanthan macromoleculars but the detailed mechanism related to the balance and interaction of these proteins is still unknown, although some similar membrane protein crystal structures have been reported (Whitfield, 2006). Additionally, according to the proteome analyses of strain *X. campestris* B100, the proteome and transcript levels of some genes were quite different, especially GumD, which suggested that post‐transcriptional regulation and a much more frequent regulation of protein expression by non‐transcriptional mechanisms than expected might exist (Hahn et al., 2022). Therefore, the translational level, assembly and run of the membrane protein machine might be the keys to mass‐producing xanthan gum.

Additionally, two promoters are located upstream of the genes *gumB* and *gumK*, respectively (Becker et al., 1998; Katzen et al., 1996). In the five engineered strains, the transcriptional levels of *gumJ* and *gumM* were all significantly upregulated, which might be due to their lower transcripts of the original control promoter *P*_*gum*_ of strain NXdPE::*gum* caused by the longest distance between their respective loci and promoters. In high‐yield xanthan‐producing strains NXG*‐P*_916_ and NXG*‐P*_828_, genes *gumBCDJLM* were significantly upregulated compared with strain NXdPE::*gum*, while expression levels of most genes in strains with low‐yield xanthan gum (NXG*‐P*_5218_, NXG*‐P*_5286_, and NXG*‐P*_*tac*_) were lower than those from other strains with high‐yield xanthan gum. Therefore, replacing the first promoter with endogenous promoters *P*_916_ and *P*_828_ facilitated the transcription of the entire *gum* operon, thus improving the synthesis of xanthan gum.

Among 12 *gum* genes, *gumFGL* encoding acetyltransferase could be knocked out without affecting polysaccharide production (Hassler & Doherty, 1990; Wu et al., 2019); gene *gumBCEJ* and *gumM* were lethal to the wild‐type strain (Becker et al., 1998); and the other glycotransferase coding genes were also vital since the inactivation would change the primary structure of xanthan gum (Katzen et al., 1998). Therefore, for xanthan biosynthesis in the *Sphingomonas* strain, the expression of *gumFGL* probably not the key factor, while the efficient transcription, translation of other genes and the correct assembly of protein complexes GumBCDEJ might be essential.

To further improve xanthan gum yield, genes *gumBCDE* were overexpressed in strains NXdPE::*gum*, NXG*‐P*_828_ and NXG*‐P*_916_ by introducing a plasmid pBT*gumBCDE*. Results showed that the transcriptional levels of these four genes increased significantly, especially the first gene *gumB*, which was upregulated by 5.7 to 20.3 times (Figure S1), but the yield decreased (Figure 4A). Such as, the yield of strain NXG*‐P*_916_ (pBT*gumBCDE*) was 3.48 ± 0.47 g/kg, which was 63.3% lower than that of strain NXG*‐P*_916_. Additionally, the whole operon *gum* was also overexpressed by introducing plasmid pBBR*gum* into the chassis NXdPE, but the yield of xanthan gum was only 1.92 ± 0.40 g/kg (Figure 4A). These results suggested that the ratio of each enzyme in the polymerisation and export protein complex was crucial, and the overexpression of genes *gumBCDE* might have affected the formation of complex. In sum, the engineered strain NXG*‐P*_916_ produced the highest yield of xanthan gum, but the transcriptional level of each *gum* gene still needs to be regulated in future work.

### Utilisation of glucose or xylose to produce xanthan gum by the engineered strain

**FIGURE 5:**
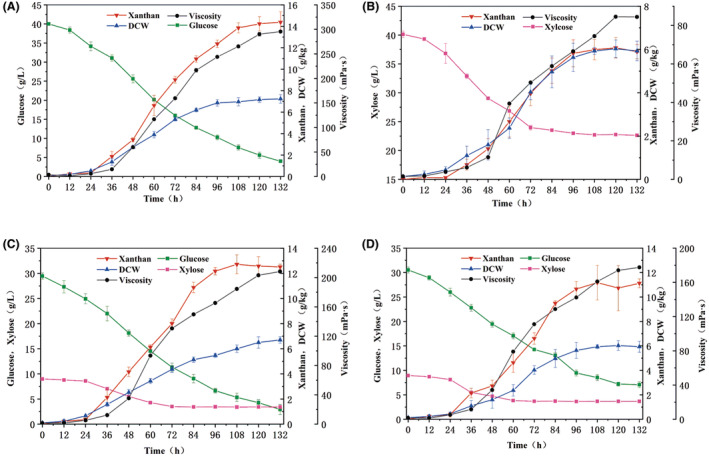
Performance of batch fermentation using glucose (A), xylose (B), the total hydrolysate (C) and simulated hydrolysate (D) as the carbon sources in a 5‐L fermenter.

Glucose and xylose, as main components of lignocellulosic hydrolysate, could be co‐utilised by the original strain NX02 (Wu et al., 2021), and their kinetic conversion processes to produce xanthan gum by the engineered strain NXG*‐P*_916_ in 5 L fermenters are shown in Figure 5A,B. At the end of glucose fermentation, the yield of xanthan gum was 14.39 ± 0.96 g/kg, DCW was 7.23 ± 0.40 g/kg, and residual glucose was 4.00 ± 0.40 g/L (Figure 5A). At the end of xylose fermentation, the yield of xanthan gum was 6.08 ± 0.47 g/kg, DCW was 6.03 ± 0.40 g/kg, and residual xylose was 22.62 ± 0.11 g/L, which was much higher than that of glucose fermentation (Figure 5B). For cell growth, DCW from xylose fermentation was slightly lower than that from glucose fermentation, which might be related to the different pathways of energy synthesis. For polysaccharide production, the yield from glucose fermentation was much higher than that from xylose fermentation, which is consistent with the original strain NX02 (Wu et al., 2021). The UDP‐sugar precursors for xanthan gum synthesis are derived from glucose‐6‐phosphate, which could be obtained from glucose catalysed by glucokinase directly. When xylose is the substrate, glucose‐6‐phosphate is obtained through the gluconeogenetic pathway, which can be divided into three sources: from xylose to the XI pathway to the PPP pathway, from xylose to the Dahms pathway to the pyruvate, and from xylose to the Weimberg pathway to the TCA cycle to phosphoenolpyruvate (Figure 1). That is, although there are three xylose metabolism pathways in an engineered strain, the synthesis of the active precursors from glucose remains the more direct route (Figure 1). Therefore, the utilisation of glucose in strain NXG*‐P*_916_ was more efficient to produce xanthan gum, which was similar to the original strain NX02 (Wu et al., 2021). From the batch fermentation profile, the yields of xanthan gum and DCW reached a stable period at about 108 h, which was longer than the original strain NX02, and the lag phase of the strain was about 24 h, which was probably because of low assembly efficiency of the membrane protein complex in the chassis strain affected the growth rate of the engineered strain and the biosynthesis rate of xanthan gum.

### Co‐utilisation of glucose and xylose from corn straw total hydrolysate to produce xanthan gum

Corn straw, as an economical and sustainable agricultural by‐product, was a promising alternative carbon source to produce xanthan gum. The total hydrolysate and simulated hydrolysate (containing 52.60 g/L glucose and 15.10 g/L xylose) were used to analyse the ability of engineered strain NXG*‐P*_916_ to convert corn straw into xanthan gum, and the time‐course profiles are shown in Figure 5C,D. When cultured in media containing total hydrolysate, glucose and xylose were consumed simultaneously. With the extension of the incubation time, the viscosity of the broth, and the yield of xanthan gum and DCW all increased gradually. At the end of the fermentation, the yields and DCW were 12.72 ± 0.75 and 6.70 ± 0.26 g/kg, the viscosity was 208.30 mPa s, and 2.93 ± 1.01 g/L glucose and 3.40 ± 0.02 g/L xylose were residual (Figure 5C). When cultured in a simulated hydrolysate medium, a similar phenomenon was found, and the yields and DCW were 11.17 ± 1.42 and 6.03 ± 0.41 g/kg, respectively (Figure 5D), which showed no significant difference from total hydrolysate fermentation. However, the yield of xanthan gum from hydrolysate was slightly lower than that from glucose fermentation, which might be due to the longer synthetic pathway from xylose to xanthan gum. What is more, residual sugar contents in the fermentation of strain NXG*‐P*_916_, especially the residual xylose, were higher than those from Sanxan production of the original strain NX02 and PHB‐defective strain NXdP. The basic fermentation characteristics of polysaccharide‐producing strains derived from strain NX02 are shown in Table S3. Compared to strains NX02 and NXdP showing high carbon conversion ability, strain NXG*‐P*_916_ containing the exogenous synthetic module of xanthan gum produced only a relative low yield of polysaccharide, which might result in the incomplete utilisation of sugars, and this low sugar conversion rate might lead to a longer fermentation time. More residual xylose might be related to the longer pathway from xylose to polysaccharide.

In the previous reports, glucose and xylose were always utilised separately. For example, when cultured in the enzymatic hydrolysate of alkali‐pretreated rice straw, 9.7 ± 0.25 g xanthan gum per 100 g of raw rice straw was produced (Jazini et al., 2017), and 8.9 g/L xanthan gum could be obtained from hydrolysis of 4% (w/v) broomcorn stem with 6% sulphuric acid (Zahra et al., 2018). Additionally, when *Melaleuca alternifolia* residue hydrolysate was the carbon source, 5.0 g/L xanthan gum was produced after 48 h of fermentation (Li et al., 2022). *Xanthomonas* sp. 629 produced 8.37 ± 5.75 g/L xanthan gum when using a fermentation medium containing a carbon source of 1.25% saccharose, 3.75% hemicellulose fractions of corncob, and salts (Jesus et al., 2023). These yields were all lower than that of strain NXG*‐P*_916_, which suggested that this strain that could co‐utilise glucose and xylose from the total hydrolysate has an application potential for xanthan gum production. However, in the original strain *X. campestris* CGMCC 15155, 28.86 ± 0.41 g/L xanthan gum also could be obtained when using 4% sucrose as a carbon source in our previous work (Wu et al., 2019). Mohsin et al. (2021) also analysed the kinetic modelling of xanthan production using different carbon sources, and xanthan gum production reached 34.06 ± 0.87 and 31.96 ± 0.62 g/L with 7% glucose and xylose as carbon sources. These original xanthan‐producing strains without characteristic of co‐utilisation glucose and xylose showed much higher yield than strain NXG*‐P*_916_, which might be because the chassis NXdPE was a Sanxan‐producing strain derivative and it possessed a less productive than the xanthan‐producing strains. Therefore, although engineered strain NXG*‐P*_916_ can effectively convert lignocellulosic biomass total hydrolysate to xanthan, it still needs to be modified to improve the yield of xanthan gum.

Carbon catabolite repression is performed through the phosphoenolpyruvate‐dependent phosphotransferase system (PTS), a common carbohydrate transport system in most bacteria (Yao & Shimizu, 2013). The simultaneous utilisation of glucose and xylose in the engineered strain NXG*‐P*_916_ was mainly due to the incomplete PTS of the original strain NX02, and the glucose metabolism is not affected because of the existence of the Entner‐Doudoroff pathway (ED), glucose isomerase and glucokinase (Wu et al., 2021). Therefore, the engineered strain NXG*‐P*_916_ with natural CCR‐negative property has a significant competitive advantage in the conversion of agricultural biomass into xanthan gum.

### The primary structure analysis of xanthan gum produced from the engineered strain NXG*‐P*_916_

To determine the type of polysaccharide produced from the engineered strain, the primary structures of polysaccharides from the original strain NX02, the wild‐type strain *X. campestris* CGMCC 15155, and the engineered strain NXG*‐P*_916_ were comparatively analysed through FT‐IR and monosaccharide analysis. The FT‐IR spectra profiles of xanthan gum from strains CGMCC 15155 and NXG*‐P*_916_ were similar (Figure S2) but much different from those of Sanxan. The main structural difference between xanthan and Sanxan was the existence of rhamnose in Sanxan (García‐Ochoa et al., 2000; Huang et al., 2016). In the FT‐IR spectrum, a band at 1055 cm^−1^ was found in Sanxan, which was the specific band of rhamnopyranoside (Kac̆uráková et al., 2000). HPLC results showed that Glu, GlcA and Man all existed in xanthan‐*P*_916_, which was consistent with the monosaccharide composition of xanthan‐wt (Figure S3). Therefore, the polysaccharide produced by strain NXG*‐P*_916_ was xanthan gum, and its primary structure was much different from Sanxan.

**FIGURE 6:**
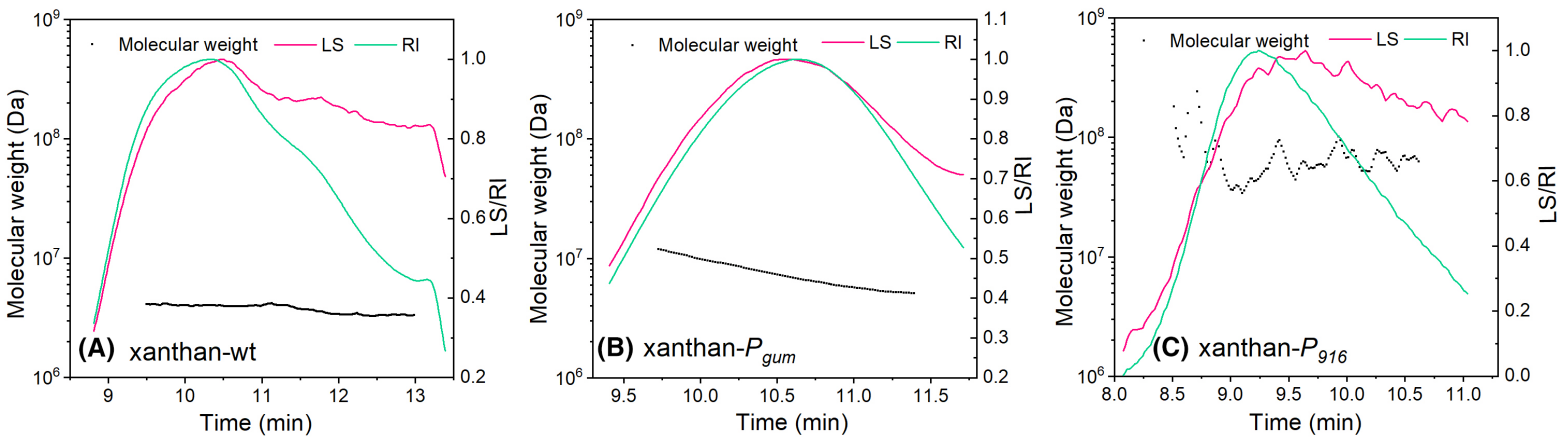
Determination of molecular weights of xanthan‐wt produced from the wild‐type *Xanthomonas* strain (A), xanthan‐*P*_*gum*_ from the engineered strain NXdPE::*gum* (B) and xanthan‐*P*_916_ from the engineered strain NXG*‐P*_916_ (C). LS, laser light scattering; RI, refractive index.

The molecular weight (MW) of xanthan gum was also measured, and the results are shown in Figure 6. Compared to the wild‐type xanthan gum produced from the original strain *X. campestris*, the MW of xanthan‐*P*_*gum*_ produced from strain NXdPE::*gum* was high, and it was 7.52 × 10^6^ Da (Figure 6B), while that of the wild‐type xanthan‐wt was 3.83 × 10^6^ Da (Figure 6A). Surprisingly, the MW of xanthan‐*P*_916_ produced from strain NXG*‐P*_916_ was 6.04 × 10^7^ Da, which was 15.8 times as much as xanthan‐wt and 8.0 times as much as xanthan‐*P*_*gum*_ (Figure 6C). Additionally, the signals of laser light scattering (LS) and refractive index (RI) did not coincide, and the polydispersity (Mw/Mn) of xanthan‐*P*_916_ was 1.06, which suggested that its molecular weight distribution was relatively uniform. Based on reports, the MW of common xanthan gum ranges from 2.0 × 10^6^ to 2.0 × 10^7^ Da (García‐Ochoa et al., 2000), while the MW of xanthan‐*P*_916_ was 3.0 times the maximum value. Therefore, xanthan‐*P*_916_ belongs to the ultrahigh molecular weight xanthan gum (greater than 2.0 × 10^7^ Da, UHMW xanthan gum).

According to the report of Galván et al. (2013), longer xanthan chains could be obtained from the strain that simultaneously overexpressed *gumB* and *gumC*. The slow growth rate might also facilitate the extension of the xanthan chain. Therefore, compared to the wild‐type strain, the growth rate of strain NXdPE::*gum* reduced, which resulted in a 2.0‐fold increase in MW. Additionally, the expression levels of *gumB* and *gumC* in the engineered strain NXG*‐P*_916_ were higher than those of strain NXdPE::*gum* (Figure 4), and the MW was further increased in xanthan‐*P*_916_. In brief, the production of UHMW xanthan*‐P*_916_ from strain NXG‐*P*_916_ was probably due to the overexpression of GumBC and the relatively slow growth rate.

## CONCLUSION

To facilitate the bioconversion of lignocellulose biomass into polysaccharide xanthan gum, an engineered strain NXG*‐P*_916_ was obtained by introducing the synthetic module of xanthan gum into the genome and optimising the promoter of operon *gum* based on a chassis NXdPE. Moreover, strain NXG*‐P*_916_ co‐utilised glucose and xylose from corn straw total hydrolysate to produce 12.72 ± 0.75 g/kg xanthan gum, which was the ultrahigh molecular weight xanthan gum with an MW of 6.04 × 10^7^ Da. This work implements the simultaneous conversion of glucose and xylose from lignocellulose biomass into xanthan gum, which resulted in a new type of UHMW xanthan gum for the first time, and it also promotes the sustainable development of the circular agricultural economy.

## AUTHOR CONTRIBUTIONS

**Mengmeng Wu:** Conceptualization (equal); funding acquisition (equal); investigation (equal); methodology (equal); writing – original draft (equal). **Zhuangzhuang Shi:** Data curation (equal); investigation (equal); methodology (equal). **Yue Ming:** Data curation (equal); formal analysis (equal). **Yufei Zhao:** Data curation (equal); formal analysis (equal). **Ge Gao:** Data curation (equal); formal analysis (equal). **Guoqiang Li:** Methodology (equal); visualization (equal); writing – review and editing (equal). **Ting Ma:** Conceptualization (equal); funding acquisition (equal); supervision (equal); writing – review and editing (equal).

## CONFLICT OF INTEREST STATEMENT

The authors declare no competing interests.

## Supporting information


Data S1:

## Data Availability

The data that support the findings of this study are available from the corresponding author.
