# Supplementary material for: The production of ultrahigh molecular weight xanthan gum from a Sphingomonas chassis capable of co‐utilising glucose and xylose from corn straw
Source: Microb Biotechnol. 2024 Jan 16;17(2):e14394. doi: 10.1111/1751-7915.14394 (PMC10884872; doi:10.1111/1751-7915.14394)
Supplement: Supplementary file 1 — Data S1: [file MBT2-17-e14394-s001.docx]

**Title:** The production of ultrahigh molecular weight xanthan gum from a *Sphingomonas* chassis capable of co-utilizing glucose and xylose from corn straw

**Running title:** Production of ultrahigh molecular weight xanthan gum

Mengmeng Wu ^#^, Zhuangzhuang Shi ^#^, Yue Ming, Yufei Zhao, Ge Gao, Guoqiang Li*, Ting Ma*

**Affiliations**

Key Laboratory of Molecular Microbiology and Technology, Ministry of Education, College of Life Sciences, Nankai University, Tianjin, 300071, China

Table S1 Primers for the construction of the engineered strain or plasmid in this work

| Primers | Sequence (5’→3’) |
| --- | --- |
| Primers for the third segment *gumBCDE* of operon *gum* (5.20 kb) | |
| 1XMU | gatgaacgacgggcagggccaacgccgtcaaaaaaatg |
| 1XML | gtctgatgcgcgacgatccaccacagcgccgatgtcttga |
| 1POU | catgcgatcgaccttgcggcaagctattctcatgtttga |
| 1POL | cttcttctcgatctcgggcaagctcgaattaaaggatctagg |
| 1SU | cctagatcctttaattcgagcttgcccgagatcgagaagaag |
| 1SL | catttttttgacggcgttggccctgcccgtcgttcatc |
| 1XU | tcaagacatcggcgctgtggtggatcgtcgcgcatcagac |
| 1XL | tcaaacatgagaatagcttgccgcaaggtcgatcgcatg |
| Primers for the second segment *gumEFGHI* of operon *gum* (4.60 kb) | |
| 2POU | catgcgatcgaccttgcggcaagctattctcatgtttga |
| 2POL | agaccagaaattccacggcatgctcgaattaaaggatctagg |
| 2SU | cctagatcctttaattcgagcatgccgtggaatttctggtct |
| 2SL | agccgcagacaaacca ccacagcgccgatgtcttga |
| 2XMU | tcaagacatcggcgct gtggtggtttgtctgcggct |
| 2XML | gtctgatgcgcgacgatccacggtgttggggaagtcatgg |
| 2XU | ccatgacttccccaacaccgtgg atcgtcgcgcatcagac |
| 2XL | tcaaacatgagaatagcttgccgcaaggtcgatcgcatg |
| Primers for the third segment *gumJKLM* of operon *gum* (5.20 kb) | |
| 3POU | catgcgatcgaccttgcggcaagctattctcatgtttga |
| 3POL | cagtcgtagcgcgccacatacgctcgaattaaaggatctagg |
| 3SU | cctagatcctttaattcgagcgtatgtggcgcgctacgactg |
| 3SL | cacctgatcgagcatgccgctcaacaacgccgcatcga |
| 3XMU | tcgatgcggcgttgttgagcggcatgctcgatcaggtg |
| 3XML | gtctgatgcgcgacgatccatgccaacaacgacaccaccaa |
| 3XU | ttggtggtgtcgttgttggcatgg atcgtcgcgcatcagac |
| 3XL | tcaaacatgagaatagcttgccgcaaggtcgatcgcatg |
| Primers for gene *SsH* knockout (SU/SL/XU/XL) and identification (1/2) | |
| *ssH*SU | gagataagagctccccttgcggatcgtgattct (*Sac*Ⅰ) |
| *ssH*SL | actgaacgatggccttctccggcaccggcaggttttcc |
| *ssH*XU | ggaaaacctgccggtgccggagaaggccatcgttcagt |
| *ssH*XL | gctgtcctctagaccgcctatgcagatacgct (*Xba*Ⅰ) |
| *ssH*-1 | ccaaccgcacgatcaaa |
| *ssH*-2 | gttcgccttcgcatagagc |
| Primers for gene *SsQ* knockout (SU/SL/XU/XL) and identification (1/2) | |
| *SsQ*SU | gagatccttaattaacgcagcccgccttggtaa (*Pac*Ⅰ) |
| *SsQ*SL | attgtatcgcccagctcggcccctcgctttatccggttgt |
| *SsQ*XU | acaaccggataaagcgaggggccgagctgggcgatacaat |
| *SsQ*XL | gcaggactctagagcgcagacgatcctcgatt (*Xba*Ⅰ) |
| *SsQ-*1 | caggatccgccgctcata |
| *SsQ-*2 | gcagaacgcgaagagctatctc |
| Primers for operon *SsGCD* knockout (SU/SL/XU/XL) and identification (arm1/arm2/out1/out2) | |
| *SsGCD*SU | gagcctcttaattaacaacccgctgacgatctggt (*Pac*Ⅰ) |
| *SsGCD*SL | gaccggagcagaatggcgagcgacggcgatgcagttc |
| *SsGCD*XU | gaactgcatcgccgtcgctcgccattctgctccggtc |
| *SsGCD*XL | gcaatgatctagacgtgacccggccctatgac (*Xba*Ⅰ) |
| *SsGCD*arm 1 | caatgcgcgccatcataag |
| *SsGCD*arm 2 | gagcgccgatccttaatcac |
| *SsGCD*out 1 | ctgcggcttggatacaccc |
| *SsGCD*out 2 | cggccgcaactatctcgttt |
| Primers for pBC*P_828_rfp* construction | |
| 828SU | gtgctatggtaccgtgcgcgccttcgatcct (*kpn*Ⅰ) |
| 828SL | cctcctcgcccttggatcccatgtttcactccctcatgacttcg |
| 828-mrfp-P1 | cgaagtcatgagggagtgaaacatgggatccaagggcgaggagg |
| mrfp-P2 | ggcgcgtctagattacttgtacagctcgtccatgcc (*xba*Ⅰ) |
| Primers for pBC*P_916_rfp* construction | |
| 916SU | gaggaggggtaccgcgcagcttcaccgacatc (*kpn*Ⅰ) |
| 916SL | cctcctcgcccttggatcccatcctttgtcgcgcttataagcc |
| 916-mrfp-P1 | ggcttataagcgcgacaaaggatgggatccaagggcgaggagg |
| mrfp-P2 | ggcgcgtctagattacttgtacagctcgtccatgcc (*xba*Ⅰ) |
| Primers for pBC*P_1218_rfp* construction | |
| 1218SU | gagagagggtacccgcgctccagcacatagag (*kpn*Ⅰ) |
| 1218SL | cctcctcgcccttggatcccatggggttgcagggacgtcat |
| 1218-mrfp-P1 | atgacgtccctgcaaccccatgggatccaagggcgaggagg |
| mrfp-P2 | ggcgcgtctagattacttgtacagctcgtccatgcc (*xba*Ⅰ) |
| Primers for pBC*P_5194_rfp* construction | |
| 5194SU | ggagaggggtaccacgatggcagactggcgg (*kpn*Ⅰ) |
| 5194SL | cctcctcgcccttggatcccatcaatcagaacctttcactggaa |
| 5194-mrfp-P1 | ttccagtgaaaggttctgattgatgggatccaagggcgaggagg |
| mrfp-P2 | ggcgcgtctagattacttgtacagctcgtccatgcc (*xba*Ⅰ) |
| Primers for pBC*P_5217_rfp* construction | |
| 5217SU | ggagaagggtacccgcggatctcatcgacatc (*kpn*Ⅰ) |
| 5217SL | cctcctcgcccttggatcccatgacgtgggcgatcctttga |
| 5217-mRfp-P1 | tcaaaggatcgcccacgtcatgggatccaagggcgaggagg |
| mrfp-P2 | ggcgcgtctagattacttgtacagctcgtccatgcc (*xba*Ⅰ) |
| Primers for pBC*P_5218_rfp* construction | |
| 5218SU | gaggatgggtaccaccaccgatcccgacacc (*kpn*Ⅰ) |
| 5218SL | cctcctcgcccttggatcccatcgcaaaacgtccctttctt |
| 5218-mRfp-P1 | aagaaagggacgttttgcgatgggatccaagggcgaggagg |
| mrfp-P2 | ggcgcgtctagattacttgtacagctcgtccatgcc (*xba*Ⅰ) |
| Primers for pBC*P_5286_rfp* construction | |
| 5286SU | gaggggaggtaccgtgaagacgcccgcctat (*kpn*Ⅰ) |
| 5286SL | cctcctcgcccttggatcccatctgacactcctaaacgctttatg |
| 5286-mRfp-P1 | cataaagcgtttaggagtgtcagatgggatccaagggcgaggagg |
| mrfp-P2 | ggcgcgtctagattacttgtacagctcgtccatgcc (*xba*Ⅰ) |
| Primers for pBBR*gum* construction |  |
| gum-pBBR U | agaacatggtggatggcacgtgagtcgtattacgcgcgct |
| gum-pBBR L | gacactgcgaccacttccgtgcttggcgtaatcatggtcata |
| 1gumU | tatgaccatgattacgccaagcacggaagtggtcgcagtgtc |
| 1gumL | tagctgaacgaaaaagggccagaatcatctggggtacatgcttg |
| 2gumU | caagcatgtaccccagatgattctggccctttttcgttcagcta |
| 2gumL | cggtcaagtgatgagctcgcagtttatgcgggcctatgttc |
| 3gumU | gaacataggcccgcataaactgcgagctcatcacttgaccg |
| 3gumL | ggtcaagacgcgacggatccagcgcgaactgttcctgtt |
| Primers for RT-qPCR analysis | |
| 16sq1 | ggcggttcctttagagtaccc |
| 16sq2 | aaccttaccagcgtttgacatg |
| mrfpq1 | gccaagctgaaggtcaccaa |
| mrfpq2 | gggccgtctgaagggaagtt |
| gumBq1 | agcccgaataccgtcttgc |
| gumBq2 | tcctgcacgaataccgaaatc |
| gumCq1 | gccgccaagatcgccaatac |
| gumCq2 | ctcagccagaaactgtgtcgc |
| gumDq1 | cgcgtgttcgacctgaccat |
| gumDq2 | aaccacgcgacacctgctca |
| gumEq1 | cgggctaggcttgatcgtga |
| gumEq2 | gcgcattgacgaagaagctc |
| gumFq1 | tggtcaccgtgattggctac |
| gumFq2 | catgtgctggagcggaaac |
| gumGq1 | tctggtggcttacggctactgg |
| gumGq2 | caaggccgggagcacatacaag |
| gumHq1 | gtccgccagttccatccgt |
| gumHq2 | acgatagccgatgcgagtga |
| gumIq1 | cgccaacaccaacccgtatc |
| gumIq2 | gccggtcagctgcaacttca |
| gumJq1 | cgtccaggcgaaacatgtca |
| gumJq2 | ccaacacctgcgaacccaca |
| gumKq1 | acgacggtgcatccattcaa |
| gumKq2 | cacgttgatggtgctcagcc |
| *gumL*q1 | ctgctgggtgcggaattt |
| *gumL*q2 | ggtgccgtggtagtagtcgt |
| *gumM*q1 | ctcaacggcaccgacctga |
| *gumM*q2 | cgcaaattcgccataccca |

Table S2 Gene annotations, upstream sequence of initiation codon (ATG), and predicted promoter sequence (underline) by using BDGP Neural Network Promoter Prediction V2.2

| Gene | Annotation | Upstream sequence of initiation codon (ATG) and predicted promoter sequence (underline) by using BDGP Neural Network Promoter Prediction V2.2 |
| --- | --- | --- |
| *orf828* | Hypothetical protein | CGGCTGATGGCGTTGCCGATTCTCGTCGATCTGCGCAACATCTATCCCCCCGAGGAAGCACTGGCGGCGGGATTCTCCTACACGTCGGTCGGCCGCAGCGCGGTTTCGGGGGATTTTGCCGCAGTGCAACCAAAGATGGCGGAGGACGCGGCGACAGGGTAATAGGACGGCGGAGAAACGGAACCTGAGTCGTGCATAACCGTTGGTCGCCTGAATCTCTGCTGCACTGCGATATGACACTTGCGTAATCAATTCATGTATGGCATTGCAGCATCAATCGAGCACGGCACACGAAGTCATGAGGGAGTGAAAC**ATG** |
| *orf916* | ATP-dependent Clp endopeptidase proteolytic subunit CppP | GCGCGGCCGGACGGCTCCGCCTCCAAGCTGGCGACCTATCGCGACGGGTGGCGGATCTTCCGGACGATCGGCACGCTGTATCGTATCGAGCGGCCGGTGCTCTATTTCGGCGGGATCGGCGCCGTGCTGGTGCTGGCGGCGGTGATCCTGGCGCTGCCGCTGCTGTTCACCTGGCTCCAGACCGGGCTGGTTCCCCGCTATCCGACCGCGGTGCTGGCGACCGGCCTTACCATCGTCGCCTTCCTCAGTTTCGCCTGCGGCCTCATCCTCGACACGGTGGTGCACGGGCGGCGCGAGATGCGGCGGATCGCCTATCTTTCGCATGCTGCGCCGGGCGCGGCCGACGCCCGAAGCGAGGCCCCTTGAAGCCGCCCCGCTTTCACCCGATGTAGGGACACGGCAAAAGGCTTATAAGCGCGACAAAGG**ATG** |
| *orf1218* | Outer membrane protein beta-barrel domain | GTTCGCCCGCCAGATATTGCCGCTCGAACTCCTTGTTCTCGCCGACATAGCTGGAGATCATCTTCTTGATCTGGCGCGTGCGGAGCAGCTTGCCGAGCCCCTCGCCATCGATGCCGGCATTGTTGGAGGCGACGGTGATGTCTTTGGTGCCGGCGGCCTGGATCGCGTCGATCAGCCGTTCGGGGATACCGCAAAGGCCGAAGCCGCCCGCGCAGATCGTCATGCCGTCGAACAGCAGGCCATCGAGGGCGCTCGCTGCGTCAGGGTAAAGCTTGTTCATCCTCGGTCCTCCCCAAGTGCGCGGCTTTGCGTTCGCATAGGCGCGGCCGGCGGCATGGGTCAATCGCGCTCTCCTGCATTCGTCATTGCGCGAACTGCACGCCGCGAGATGACGTCCCTGCAACCCC**ATG** |
| *orf5194* | CarD family transcriptional regulator | ACGACGCGATGCTGTTCGCGCGCTGAGTCAAAGTCGGCCCGGCGGACGCCAATCAAGGCGGGCTGGCAATTTTATGACGAATCTGCTATACGGGTTGCCGATGCGAACCCTTGTCCGCACCCCAAGCCATATCCTGAGTGGAGAGATCTCGACGTAACGGACGCACCAAGAACCGGCGGCCGACACCGGCAAGCCCCGTGGGGATCTGCGTCCACTTTTTCCAGTGAAAGGTTCTGATTG**ATG** |
| *orf5217* | Fructose 1,6-bisphosphatase II | CGCGGATCTCATCGACATCGCCCGCGGCGAATATGGCCCCGCCTTCGCGATGCCGGTCGATGCGCTCGCCGAACCGCCGGTCGCCTCCAGCGCCGTACGTCGCGGCCGCGCCTATCTGCGCTTCACCGTCGTCGACAAGCCGGGCGTGCTCGCCGAGATCGCCGCTGCGATGCGCGATTCGGGCGTGTCGATCGAAAGCCTCATCCAGCGCGGTACCGCGCCCGATGGCTCCGCGATCATCGCGATCGTCACCCATGCCGGCCCAGAGCGCGCGGTGACGGATGCGCTGGAACGGCTGCGGGGGTCGGCCAGCCTGACGGGTGAGCCGATGCTGATGCACATTCTCGATCTCTGAGGCACGTTTTAGCTGCATTGCAGCAAAGCCGTTCGCACCGTTCTCGACAACGCCATATCGGGCGCTTATCGCCGGATCATTCGTGCCTCTTCTCAAAGGATCGCCCACGTC**ATG** |
| *orf5218* | OmpA-family protein | CATCAAGGATCTCGACAAGGTCTACAAGCTGGACGAGCTGGTAAAGGGCGACGCGATCTTTGCGGCAACCGGCGTCACCGACGGTTCGCTGCTCGAAGGCGTCAAGCGTCGCAAGGGCTGCATGACCACCGACACGGTGGTCATGCGCGCAAGCTCCGGCACCGTCCGCTGGGTCAAGGGCGAGCACCGCAAGTTCAAGTGATGGTCGGGCGCCGGACAAGCACTTCGCTTGATCGGCGCTCTGCCCCGGTTCGTCTCTCGGCTGCGAACGCAACCCTTTTCTGTCAACCTCGTTGTTTCAGGTTCATGCAACTTCGATCAATAGCGCGGCGTTGGGTTGTTCGTTCCTCAAGAGGGAAAGAAAGGGACGTTTTGCG**ATG** |
| *orf5286* | Phasin family protein | ACGGCTGAAAGCGCTGGAAGATGCGCCGGGGAGCTATGTGCGCGCGCGTTAGGATACGCCCCGGTTACGGGATGATGACCCTGATGTGACTTTATGCTGCACTGCACAAATTCGCTTGCAATGCTTGATCTGCAGCCCTATTTGTGCGATGCAGCATAAAGCGTTTAGGAGTGTCAG**ATG** |

Table S3 The basic fermentation characteristics of polysaccharide producing strains derived from the original strain NX02. The initial sugar concentration is 40 g/L. “-” indicates no test.

| Strain | Genotype | Product | Carbon source | Yield of EPS | Yield of PHB | Residual sugar | Fermentation time | Reference |
| --- | --- | --- | --- | --- | --- | --- | --- | --- |
| NX02 | Wild-type | Sanxan PHB | Glucose | 14.88 ± 0.83 g/L | 6.08 ± 0.23 g/L | 0 | 80 h | Wu et al., 2016 |
|  |  |  | Glucose | 12.4 ± 0.61 g/Kg | - | 0 | 84 h | Wu et al., 2021 |
|  |  |  | Xylose | 8.13 ± 0.71 g/Kg | - | 0 | 84 h |  |
|  |  |  | Corn straw hydrolysate (G:X=3.48:1) | 13.10 ± 0.35 g/Kg | - | 0 | 84 h |  |
| NXdP | NX02 (∆PHB) | Sanxan | Glucose | 21.20 ± 0.38 g/L | 0 | 0 | 80 h | Wu et al., 2018 |
| NXG-*P_916_* | NX02 (∆PHB∆ss*HQGCD*:: *P_916_gum*) | Xanthan | Glucose | 14.39 ± 0.96 g/Kg | 0 | 4.00 ± 0.40 g/L | 132 h | This work |
|  |  |  | Xylose | 6.08 ± 0.47 g/Kg | 0 | 22.62 ± 0.11 g/L | 132 h |  |
|  |  |  | Corn straw hydrolysate  (G:X=3.48:1) | 12.72 ± 0.75 g/Kg | 0 | Glu: 2.93 ± 1.01 g/L  Xyl: 3.40 ± 0.02 g/L | 132 h |  |


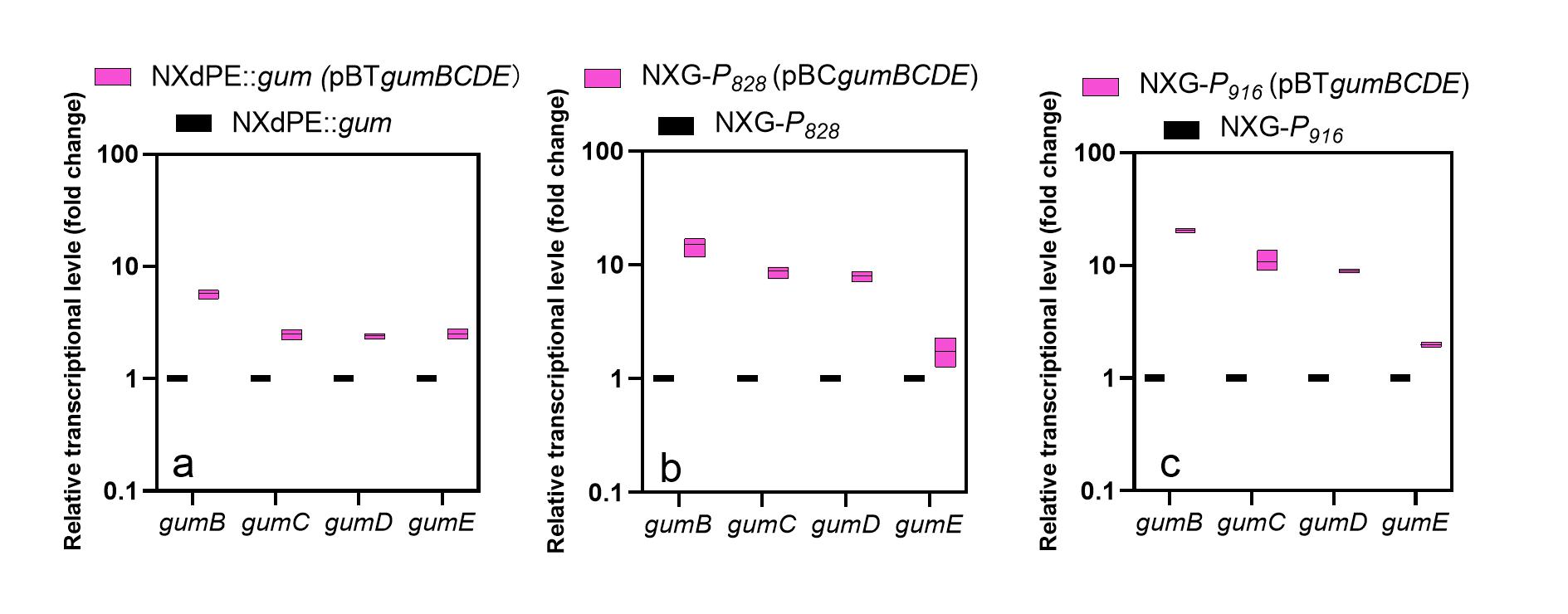


Fig. S1 Relative transcriptional level of genes *gumB*, *gumC*, *gumD*, and *gumE* in strains NXdPE::*gum* (pBTgumBCDE) (a, NXdPE::*gum* as acontrol), NXG*-P_828_* (pBTgumBCDE) (b, NXG*-P_828_* as acontrol), and NXG*-P_916_* (pBTgumBCDE) (c, NXG*-P_916_* as acontrol).


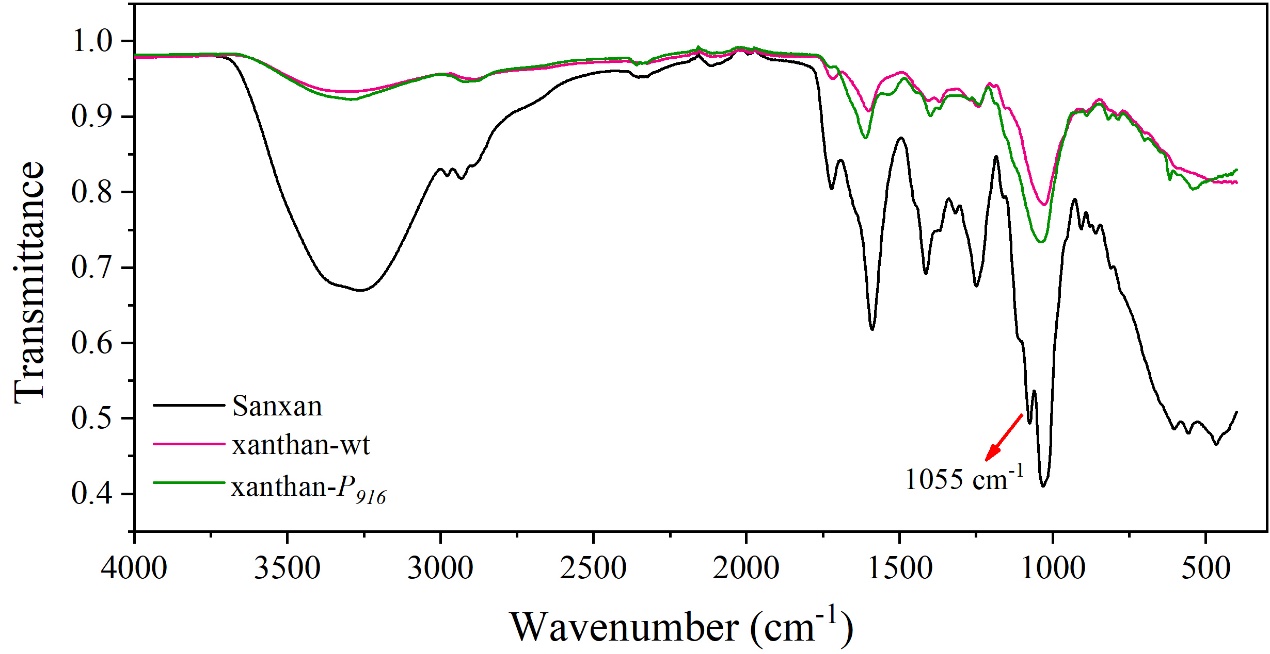


Fig. S2 FT-IR spectrums of polysaccharide Sanxan, xanthan-wt, and xanthan-*P_916_*.


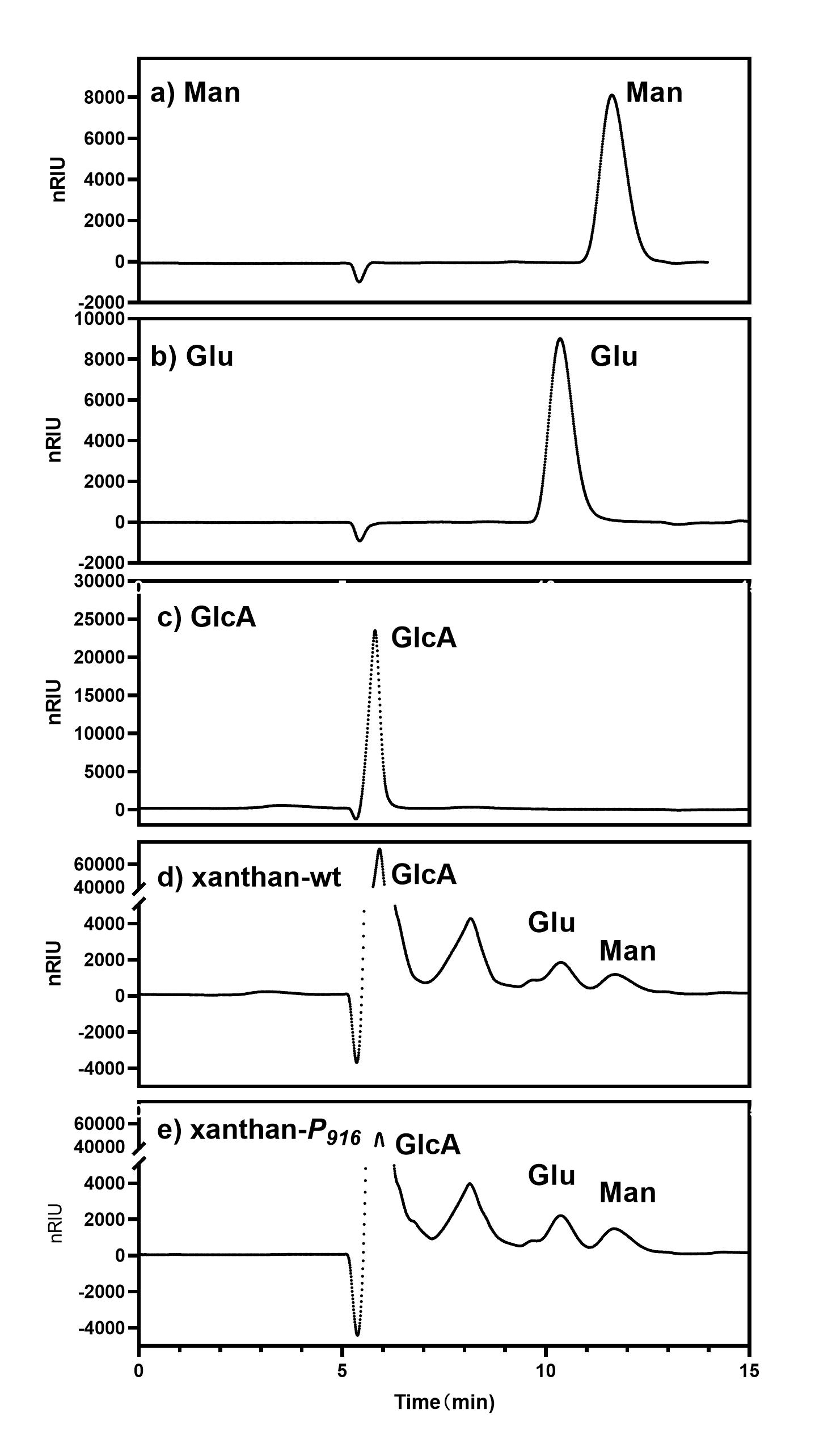


Fig. S3 Monosaccharide analysis of xanthan-wt and xanthan-*P_916_*. a，standard mannose (Man); b, standard glucose (Glu); c, standard glucuronic acid (GlcA); d) xanthan-wt; e) xanthan-*P_916_*
